# The Triad of NF-κB, HIF-1α, and Oxidative Stress in Hepatocellular Carcinoma: Pathogenesis, Clinical Challenges, and Therapeutic Potential of CIGB-552 in Liver Transplantation

**DOI:** 10.7150/ijbs.137642

**Published:** 2026-08-12

**Authors:** Xufan Cai, Muqiong Xing, Qianrang Lu, Julio R. Fernández Massó, Brizaida Oliva Arguelles, Maidel Carpio Alvarez, Ming Chen, Qi Ling

**Affiliations:** 1 Department of Hepatobiliary and Pancreatic Surgery, The First Affiliated Hospital, Zhejiang University School of Medicine, Hangzhou, 310058, China.; 2 NHC Key Laboratory of Combined Multi-Organ Transplantation, Hangzhou, 310058, China.; 3 State Key Laboratory for Diagnosis and Treatment of Infectious Diseases, National Clinical Research Centre for Infectious Diseases, Collaborative Innovation Centre for Diagnosis and Treatment of Infectious Diseases, The First Affiliated Hospital, Zhejiang University School of Medicine, Hangzhou, 310058, China.; 4 Pharmaceutical Department, Center for Genetic Engineering and Biotechnology, Havana, 10100 Cuba.; 5 Lymphoma & Genomics Research Program, Institute of Oncology Research, Bellinzona, 6500 Svizzera, Switzerland.; 6 Department of Bioinformatics, School of Life Sciences, Zhejiang University, Hangzhou, 310058, China.; 7 State Key Laboratory of Advanced Drug Delivery and Release Systems, College of Pharmaceutical Sciences, Zhejiang University, Hangzhou, 310058, China.

**Keywords:** hepatocellular carcinoma, NF-κB, HIF-1α, oxidative stress, CIGB-552, COMMD1, liver transplantation, immunotherapy

## Abstract

Hepatocellular carcinoma (HCC) represents a formidable oncological challenge characterized by complex molecular pathogenesis and limited therapeutic outcomes, particularly in the context of liver transplantation. As the sixth most commonly diagnosed cancer and the third leading cause of cancer-related mortality worldwide, HCC poses significant clinical challenges that demand innovative therapeutic approaches. Central to HCC development and progression is a pathogenic triad comprising nuclear factor-kappa B (NF-κB), hypoxia-inducible factor-1α (HIF-1α), and oxidative stress-three interconnected pathways that drive inflammation, angiogenesis, metabolic reprogramming, and cell survival. This comprehensive review examines the molecular mechanisms underlying this triad in HCC pathogenesis across different etiological contexts, including viral hepatitis and non-alcoholic fatty liver disease (NAFLD)/non-alcoholic steatohepatitis (NASH). We critically analyse the unique clinical challenges posed by HCC in liver transplantation recipients, particularly the paradoxical requirement for immunosuppression alongside antitumor immunity, and constraints surrounding immunotherapy application. Furthermore, we present CIGB-552, a novel peptide therapeutic targeting COMMD1 (Copper Metabolism MURR1 Domain-containing protein 1), as a promising dual-function agent capable of simultaneously disrupting the pathogenic triad through NF-κB inhibition, HIF-1α suppression, and strategic modulation of oxidative stress via SOD1 regulation. The multimodal mechanism of CIGB-552 offers a theoretically rational therapeutic approach for HCC management in both pre-transplant and post-transplant settings. Clinical validation in the transplantation setting is required.

## Introduction

Primary liver cancer, predominantly HCC, constitutes a formidable burden on global health systems, representing the sixth most commonly diagnosed cancer and third leading cause of cancer death worldwide 1, 2. Incidence rates continue rising in Western countries, driven by the increasing prevalence of NAFLD and NASH 3, 4. Despite advances in surgical techniques, locoregional therapies, and systemic treatments, HCC prognosis remains dismal, with five-year survival rates below 20% in advanced stages, primarily due to aggressive tumor biology, inadequate early screening, and frequent late-stage diagnosis when curative interventions are no longer feasible 5-7.

Within this complex microenvironment, three molecular players have emerged as central conductors of the malignant orchestra: Nuclear Factor-kappa B (NF-κB), Hypoxia-Inducible Factor-1α (HIF-1α), and Oxidative Stress 8. NF-κB acts as master regulator of inflammation and cell survival, often constitutively activated in HCC. HIF-1α orchestrates cellular response to hypoxia, driving angiogenic switch and metabolic reprogramming necessary for tumor expansion and promotes therapeutic resistance 9, 10. Oxidative stress, characterized by imbalance between ROS production and antioxidant defenses, serves as both a damaging agent to DNA and a crucial second messenger activating these transcription factors, forming synergistic network that creates a tumor-permissive microenvironment, promotes genomic instability, and confers resistance to conventional therapies 11.

The therapeutic landscape for HCC has evolved significantly in recent years. Systemic therapies have progressed from non-specific chemotherapeutics to targeted tyrosine kinase inhibitors (TKIs) 12 and, most recently, to immune checkpoint inhibitors (ICIs) such as atezolizumab and pembrolizumab 13, 14. While these advances have improved survival, response rates remain suboptimal, and resistance inevitably develops 15. Furthermore, for patients with early-stage HCC or those successfully downstaged, liver transplantation (LT) remains gold standard 16, 17. However, LT introduces unique clinical conundrum: the need for lifelong immunosuppression to prevent allograft rejection creates permissive environment for HCC recurrence 18. Moreover, the use of potent immunotherapies in the peri-transplant period carries high risk of triggering fatal rejection 19.

Consequently, there is an urgent unmet need for therapeutic agents that can effectively target HCC tumors while simultaneously modulating the immune system in a way that circumvents graft rejection and, ideally, promotes graft tolerance. This review elucidates mechanisms of NF-κB/HIF-1α/ROS triad in HCC, critiques current immunosuppressive paradigms in transplant oncology, and provides in-depth analysis of CIGB-552, novel peptide drug with unique multimodal mechanism of action addressing these specific challenges 20.

## The Molecular Triad in HCC Pathogenesis

**Figure 1:**
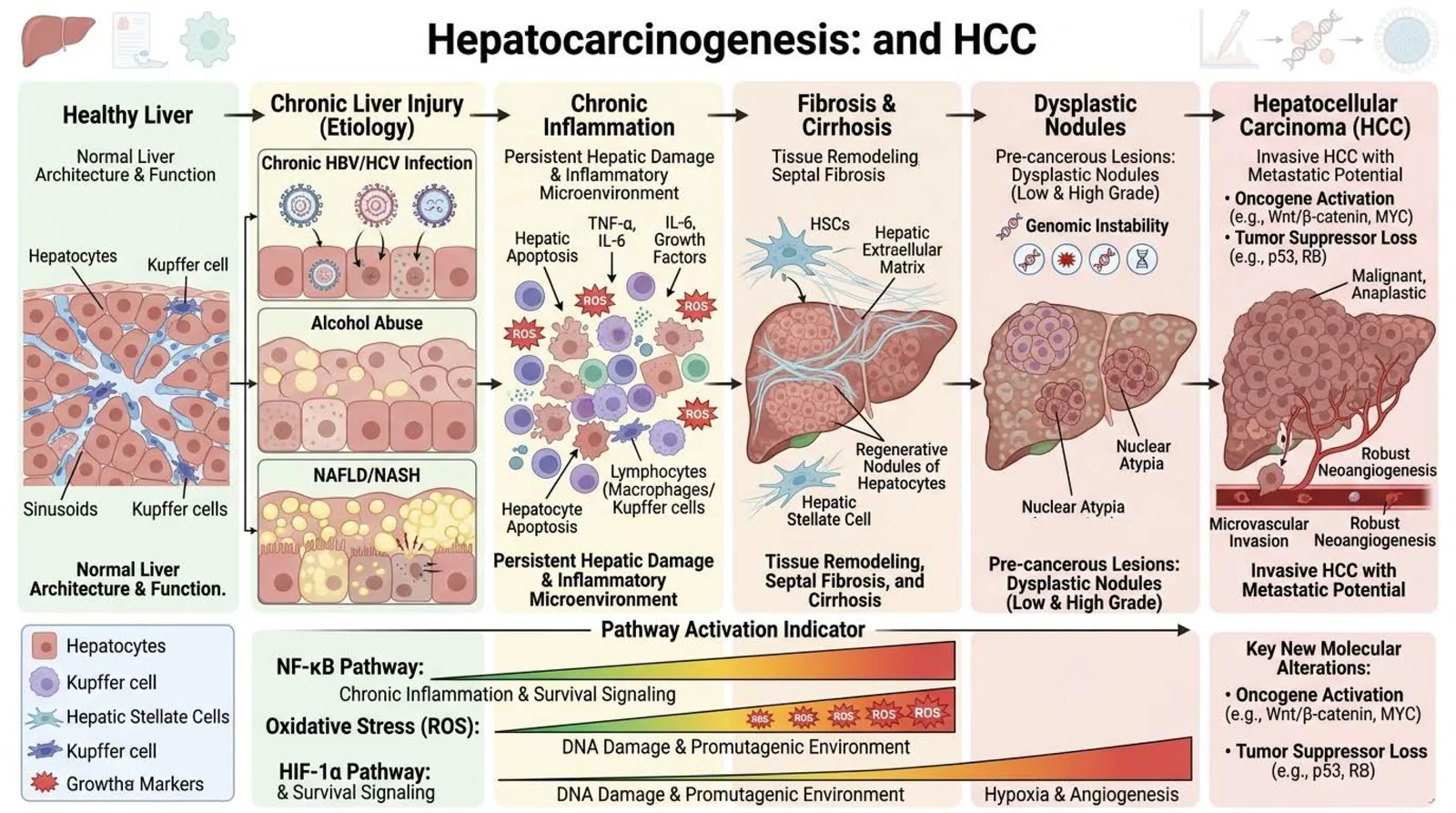
** Pathophysiological Stages of HCC Development.** This figure outlines the clinical and molecular evolution of liver cancer. It highlights the role of chronic inflammation, oxidative stress, and the activation of key signaling pathways (NF-κB and HIF-1α) in driving genomic instability and cellular atypia. The progression emphasizes the shift from regenerative nodules in cirrhosis to invasive, metastatic HCC.

The HCC represents a multistep process occurring on background of chronic liver injury rather than a singular event (Figure 1). Whether the etiology is viral (hepatitis B or C), metabolic (NAFLD/NASH), toxic (alcohol), or genetic, the common denominator remains persistent hepatic inflammation and tissue remodelling. This chronic inflammatory state creates microenvironment rich in reactive oxygen species (ROS), cytokines, and growth factors that collectively promote hepatocarcinogenesis. The molecular landscape of HCC demonstrates extraordinary heterogeneity driven by diverse etiological factors including chronic hepatitis B virus (HBV) and hepatitis C virus (HCV) infection, alcohol consumption, and metabolic dysfunction-associated steatosis liver disease (MASLD) 5.

### Oxidative Stress: The Initiator and Fuel

Oxidative stress represents a central pathogenic mechanism across the spectrum of liver diseases, from metabolic dysfunction to carcinogenesis. Allameh *et al*. 21 provided a comprehensive framework demonstrating that ROS generation disrupts hepatocellular homeostasis through multiple interconnected pathways, including mitochondrial dysfunction, endoplasmic reticulum stress, and impaired antioxidant defenses. This oxidative burden serves as a common denominator linking diverse etiologies; viral, metabolic, and toxic to progressive liver injury (Figure 1).

#### NAFLD/NASH: Oxidative Stress as Disease Driver

In NAFLD, oxidative stress emerges as a critical mediator of disease progression from simple steatosis to NASH and ultimately HCC. Chen *et al*. 22 systematically delineated how lipid accumulation in hepatocytes triggers mitochondrial dysfunction and NOX activation, generating ROS that promote lipotoxicity, inflammation, and fibrogenesis. These results were corroborated by Ashraf *et al*. 23, who demonstrated a direct correlation between oxidative stress severity, measured via pro-oxidant antioxidant balance, and liver fibrosis stage in NAFLD patients, establishing oxidative imbalance as a quantifiable disease biomarker.

Additionally, Gabbia *et al*. 24 specifically highlighted NADPH oxidases (NOX enzymes) as primary ROS sources in the NAFLD-NASH-HCC transition, emphasizing their dual role in promoting hepatic insulin resistance, necroinflammation, and pro-oncogenic signaling. The enzymatic source of oxidative stress thus represents a potential therapeutic target for interrupting disease progression. The transition from NAFLD to NASH to HCC represents a paradigm where metabolic, inflammatory, and oxidative processes converge, with ROS serving both as effectors of tissue damage and signaling molecules that perpetuate disease progression.

#### Obesity-Associated HCC

Brahma *et al*. 25 extended these findings to hepatocarcinogenesis, identifying obesity as a metabolic context where oxidative stress drives HCC development through distinct mechanisms: chronic low-grade inflammation, adipokine dysregulation, and lipotoxicity-induced DNA damage. The authors emphasize the therapeutic challenge posed by the heterogeneity of ROS sources in obesity-related liver cancer, necessitating multimodal antioxidant strategies rather than single-target approaches.

#### Alcohol-Related Liver Disease

Petagine *et al*. 26 investigated oxidative stress in alcohol-related liver disease using cellular models, demonstrating ethanol-induced ROS generation and its downstream effects on hepatocyte viability and function. Notably, this study validates nanoformulations as protective agents against alcohol-induced oxidative damage, illustrating the translational potential of antioxidant interventions in modulating ROS-mediated liver injury.

#### Viral associated HCC

Hepatitis B virus X protein (HBx) induces mitochondrial dysfunction by disrupting the mitochondrial membrane potential and increasing mitochondrial permeability transition, leading to elevated reactive oxygen species (ROS) production which can increase mutation frequency leading to HCC 27. Similarly, Hepatitis C virus (HCV) core proteins activate NADPH oxidases (NOX enzymes), particularly NOX1 and NOX4, generating robust ROS in hepatocytes 28.

Collectively, these studies established oxidative stress as a unifying pathogenic mechanism across liver disease etiologies. This positions ROS-modulating therapies as rational approaches for preventing or reversing liver disease across diverse clinical contexts.

Antioxidant therapies for cancer represent a complex and often contradictory area of research. The central premise is challenged by evidence that antioxidants can also protect tumor cells 29. The use of high-dose coenzyme Q10 (CoQ10) supplementation significantly improves liver steatosis, endothelial function, vascular compliance, and myocardial performance in patients with MASLD. This randomized, double-blind, placebo-controlled trial establishes CoQ10 as a clinically viable mitochondrial-targeted antioxidant. The improvement in hepatic steatosis indices suggests that CoQ10-mediated enhancement of mitochondrial electron transport chain efficiency reduces ROS generation at its source 30.

The role of ROS in HCC is paradoxical. In early hepatocarcinogenesis, high ROS levels can induce apoptosis or necrosis, acting as tumor-suppressive mechanism. However, cancer cells adapt by upregulating antioxidant defence systems, such as Nrf2/Keap1/ARE pathway. Nrf2 activation leads to expression of enzymes like superoxide dismutase (SOD), catalase (CAT), and glutathione peroxidase (GPX), allowing cancer cells to survive in high-ROS environment and exploit ROS for pro-survival signaling. This adaptation often results in redox addiction where cancer cells require certain threshold of ROS to maintain proliferative signaling but are vulnerable if that threshold is exceeded 31-33. Therapeutic strategies that deliberately induce oxidative stress represent a promising approach in cancer treatment, exploiting the unique redox vulnerability of malignant cells. By generating excessive ROS, these therapies aim to overwhelm the antioxidant capacity of cancer cells triggering cell death through apoptosis, necrosis, or ferroptosis. Importantly, this approach offers a therapeutic window for selectivity: normal tissues, which maintain lower basal ROS levels and possess robust but not overburdened antioxidant systems, can typically tolerate moderate increases in oxidative stress, while cancer cells operating near their redox limit are compelled into lethal oxidative damage 34, 35.

The oncolytic viruses are one example of this strategy. They are designed to selectively infect and kill cancer cells. Current research suggests that some of these viruses can actively promote ROS generation in HCC cells, creating a self-amplifying cycle of tumor cell death. Zhang *et al*. 36 demonstrated that a modified oncolytic vaccinia virus carrying the *Aphrocallistes vastus* lectin (oncoVV-AVL) reprograms the metabolism of HCC cells. This metabolic shift significantly increases intracellular ROS production. The elevated ROS level then promotes viral replication and induces apoptosis in HCC cells.

### NF-κB: Master Regulator of Inflammation and Survival

NF-κB is a family of transcription factors that regulates the expression of genes involved in immune and inflammatory responses, cell growth, and apoptosis. In normal hepatocytes, NF-κB activity is tightly regulated by inhibitory proteins (IκBs). However, in HCC, NF-κB is frequently constitutively activated through canonical (IKKβ-dependent) and non-canonical pathways, driven by chronic liver injury, viral infection, and inflammatory cytokines 37. Oxidative stress is a potent activator of NF-κB. ROS can activate the IκB kinase (IKK) complex, leading to phosphorylation and degradation of IκB, releasing NF-κB to translocate into the nucleus. Additionally, pro-inflammatory cytokines like TNF-α and IL-6, abundant in cirrhotic liver, activate NF-κB via respective receptors. Viral proteins, such as HBx and hepatitis C virus core protein, also directly stimulate NF-κB signaling through interactions with IKK complex components 7, 38.

In alcohol-related HCC, ethanol metabolism generates oxidative stress and LPS translocation, triggering TLR4/MyD88-dependent NF-κB signaling that promotes tumor cell invasion and angiogenesis. NF-κB pathway activation is a specific mechanism by which chronic alcohol exposure accelerates HCC progression and metastasis 39. Wu *et al*. 40 demonstrated that artesunate, an antimalarial agent with antitumor properties, inhibits HCC progression through modulation of the TLR4/MyD88/NF-κB signaling pathway. Moreover, Artemisia rupestris L suppresses HCC progression via interference with the TLR4/MyD88/NF-κB signaling axis, leading to reduced proinflammatory cytokine secretion 41.

Recent evidence demonstrates that DLGAP5 (Discs Large Associated Protein 5) governs both HCC aggressiveness and lenvatinib response through the AKT/mTOR/NF-κB axis. This work redefines NF-κB beyond its classical role as a disease driver, highlighting its function as a key determinant of targeted therapy efficacy. DLGAP5 overexpression triggers AKT/mTOR activation, subsequently amplifying NF-κB transcriptional activity and fostering a pro-survival, drug-resistant phenotype. Notably, DLGAP5 knockdown or direct NF-κB blockade restores lenvatinib sensitivity, supporting the rationale for combination strategies targeting both the kinase and the transcription factor 42.

Similarly, preclinical studies have shown that combining sorafenib with radiotherapy synergistically attenuates NF-κB activity and suppresses tumor growth in orthotopic HCC models. As a single agent, sorafenib incompletely inhibits NF-κB-mediated survival signals, while radiation-induced ROS production paradoxically activates NF-κB as a cytoprotective mechanism. The combination regimen achieves superior NF-κB suppression compared to either modality alone, providing a foundation for multimodal treatment strategies in advanced HCC 43. This confirms the therapeutic relevance of interrupting NF-κB signaling in HCC, as a pharmacologically tractable intervention point.

### HIF-1α: Orchestrating Hypoxic Response

HIF-1α serves as the master transcriptional regulator of adaptive responses under the hypoxic conditions typical of rapidly growing HCC tumors, ultimately promoting angiogenesis, epithelial-mesenchymal transition, invasion, and metastatic dissemination 44, 45. Under normoxic conditions, HIF-1α is hydroxylated by prolyl hydroxylase domain (PHD) enzymes, leading to recognition by von Hippel-Lindau (VHL) tumor suppressor and subsequent proteasomal degradation. Under hypoxia, PHD activity is inhibited, stabilizing HIF-1α. Crucially, oxidative stress also stabilizes HIF-1α. ROS can inhibit PHD enzymes or mimic hypoxia by disrupting mitochondrial electron transport chain, leading to HIF-1α accumulation even under normoxic conditions 8.

Recent investigations have revealed a paradigm-expanding finding: HCC cells express HIF-1α independently of oxygen tension during specific cell cycle phases, particularly G1/S transition. This oxygen-independent expression occurs under normoxic conditions through mechanisms involving growth factor signaling and oncogenic drivers, controlling essential metabolic pathways including glycolysis, nucleotide synthesis, and redox homeostasis. This ensures metabolic preparedness for rapid proliferation regardless of oxygen availability. This “pseudohypoxic” HIF-1α activation explains why HCC tumors exhibit glycolytic phenotypes even in well-perfused regions, and suggests that hypoxia-mimetic therapeutic strategies may be effective beyond anatomically hypoxic tumor core 46.

Competitive dynamics between hypoxia-inducible factor isoforms have been elucidated under mild chronic hypoxia conditions highly relevant to cirrhotic liver microenvironments. HIF-2α accumulates and directly interacts with c-MYC through competition with HIF-1α for limiting cofactors or binding sites, specifically driving hepatocellular proliferation distinct from HIF-1α-mediated metabolic adaptation. This isoform-specific biology has critical therapeutic implications: HIF-1α and HIF-2α are not functionally redundant but rather orchestrate distinct malignant programs, with HIF-1α primarily regulating metabolic and metastatic processes while HIF-2α controls proliferative signaling 47. Therapeutic targeting must therefore consider isoform-selective inhibition based on desired outcome, whether anti-metastatic or anti-proliferative effects are prioritized.

HIF-1α drives tumor angiogenesis by directly transactivating VEGF expression in HCC 8. Anti-VEGF therapy has become a cornerstone of systemic treatment for advanced HCC, with bevacizumab demonstrating efficacy in combination with atezolizumab as first-line therapy in the IMbrave150 trial, which showed superior overall survival compared to sorafenib 48. The rationale for VEGF blockade extends beyond anti-angiogenic effects, as VEGF signaling promotes immunosuppressive myeloid cell recruitment and resistance to immune checkpoint inhibitors, explaining the synergistic efficacy of combination therapy. However, bevacizumab carries significant risks including bleeding and gastrointestinal perforation, contraindicating its use in patients with untreated esophageal varices. Primary and acquired resistance to anti-VEGF therapy remains a major challenge, driven by compensatory angiogenic factors and HIF-1α-mediated hypoxia adaptation, necessitating next-generation combination strategies 49.

### Crosstalk: Synergistic Network

**Figure 2:**
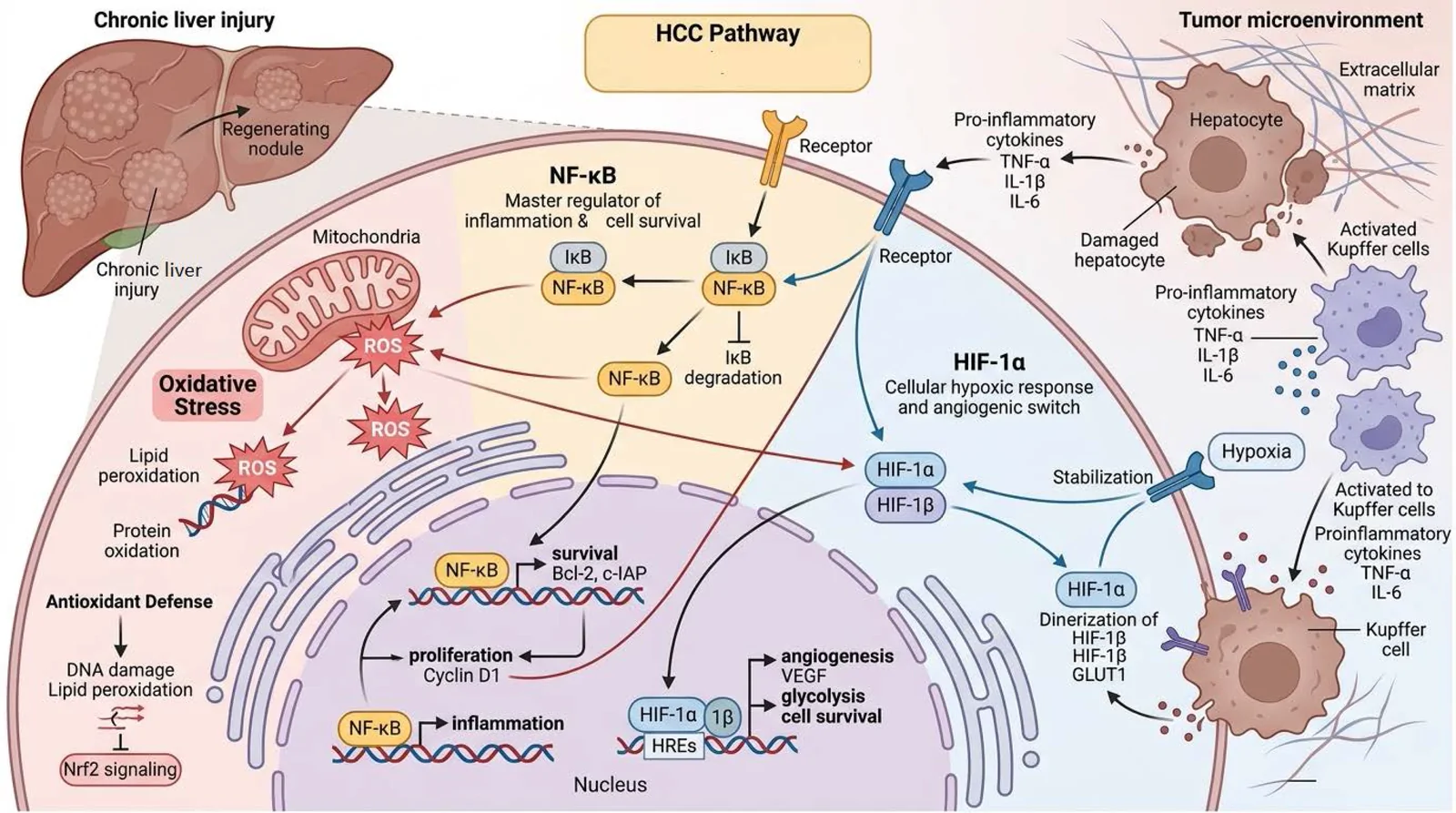
** Overview of Cellular and Molecular Mechanisms in HCC.** A summary of the “HCC Pathway” showing the roles of inflammatory signaling (NF-κB), the cellular hypoxic response (HIF-1α), oxidative stress, and the cytokine-rich tumors microenvironment in driving liver cancer progression.

The pathways governing NF-κB, HIF-1α, and oxidative stress are not isolated; they engage in extensive crosstalk that amplifies oncogenic potential 8. The molecular crosstalk between HIF-1α and NF-κB represents a critical amplification mechanism in HCC pathogenesis, operating through direct transcriptional and post-translational interactions (Figure 2). The promoter region of the HIF-1α gene contains functional NF-κB binding sites, enabling inflammatory signaling to increase HIF-1α mRNA levels and prime cells for hypoxic responses even before oxygen levels drop, a mechanism particularly relevant in cirrhotic livers where chronic inflammation and incipient hypoxia coexist 50. Conversely, HIF-1α can enhance NF-κB transcriptional activity and promote nuclear accumulation of its subunits, creating a bidirectional positive feedback loop that sustains both hypoxic adaptation and inflammatory signaling 51, 52. This reciprocal reinforcement explains why simultaneous targeting of both factors may be necessary to interrupt the self-sustaining cycle driving HCC progression, rather than inhibiting either pathway alone which permits compensatory activation through this intimate molecular cross-regulation.

Oxidative stress serves as a common upstream trigger in HCC, simultaneously activating NF-κB and stabilizing HIF-1α to create a self-reinforcing pro-tumorigenic network 53, 54. In the specific context of viral hepatitis-related HCC, ROS generation from HBV and HCV proteins disrupts mitochondrial electron transport chains and endoplasmic reticulum homeostasis, activating both NF-κB and hypoxia signaling pathways that drive malignant transformation 7, 55, 56. Furthermore, inflammatory cytokines produced by NF-κB activation, particularly TNF-α, induce further ROS production in immune cells and tumor-associated macrophages through “respiratory burst” mechanisms, creating a vicious cycle that amplifies both hypoxic and inflammatory signaling 11. This bidirectional reinforcement is exemplified by specific feedback loops that have been molecularly characterized in HCC cells. The HMGB1/NF-κB/HIF-1α loop represents a critical mechanism where high-mobility group box 1, released during cell death or actively secreted, activates NF-κB signaling, which subsequently upregulates HIF-1α transcription; HIF-1α in turn promotes HMGB1 expression, creating a self-enforcing circuit implicated in cisplatin resistance 57. Additionally, the HIF-1α/IL-8/NF-κB axis operates through HIF-1α-induced IL-8 expression, which acts as a chemokine signal that activates NF-κB and further drives HCC cell migration and invasion 58, 59. Beyond these canonical pathways, recent investigations revealed that HIF-1α transcriptionally activates HEY1, which in turn represses PINK1 to reduce mitochondrial biogenesis and reduce ROS production, demonstrating that HIF-1α can actively suppress oxidative stress as a survival strategy while simultaneously benefiting from NF-κB-mediated inflammatory adaptation 60. These interconnected loops demonstrate that oxidative stress not only initiates but perpetuates the NF-κB/HIF-1α crosstalk, establishing a robust network highly resistant to single-target interventions.

The bidirectional regulation between NF-κB and HIF-1α forms a positive feedback loop that amplifies malignant phenotypes: when NF-κB is activated by inflammatory signals or oxidative stress, it leads to increased HIF-1α expression through direct promoter binding by p50 and p65 subunits at specific sites, which promotes angiogenesis through VEGF induction and metabolic adaptation through glycolytic enzyme upregulation. Simultaneously, HIF-1α activation by hypoxia or pseudohypoxic conditions increases NF-κB activity, promoting inflammation and cell survival through anti-apoptotic gene expression 61. The temporal dynamics of this crosstalk reveal sophisticated regulatory fine-tuning, the c-Rel subunit indirectly suppresses HIF-1α through downstream microRNAs (miR-93 and miR-199a-5p), creating differential responses to acute versus prolonged hypoxia that optimize cellular adaptation 52. Necrotic debris from hypoxic HCC cells further amplifies this axis by inducing IL-1β through TLR4/TRIF/NF-κB signaling in M2-polarized tumor-associated macrophages, which in turn activates NF-κB/COX-2-mediated HIF-1α transcription, driving epithelial-mesenchymal transition and metastatic dissemination 62.

Targeting this network requires a multimodal approach capable of disrupting multiple nodes simultaneously, as inhibition of either pathway alone permits compensatory activation through the reciprocal cross-regulation. Agents that simultaneously modulate NF-κB, HIF-1α, and oxidative stress address this complexity by collapsing the self-sustaining infrastructure that supports HCC progression, offering a rational strategy particularly relevant in challenging clinical contexts such as the post-transplant setting where conventional immunotherapies are contraindicated.

## Clinical Challenges in Liver Transplantation

### Paradox of Immunosuppression

**Figure 3:**
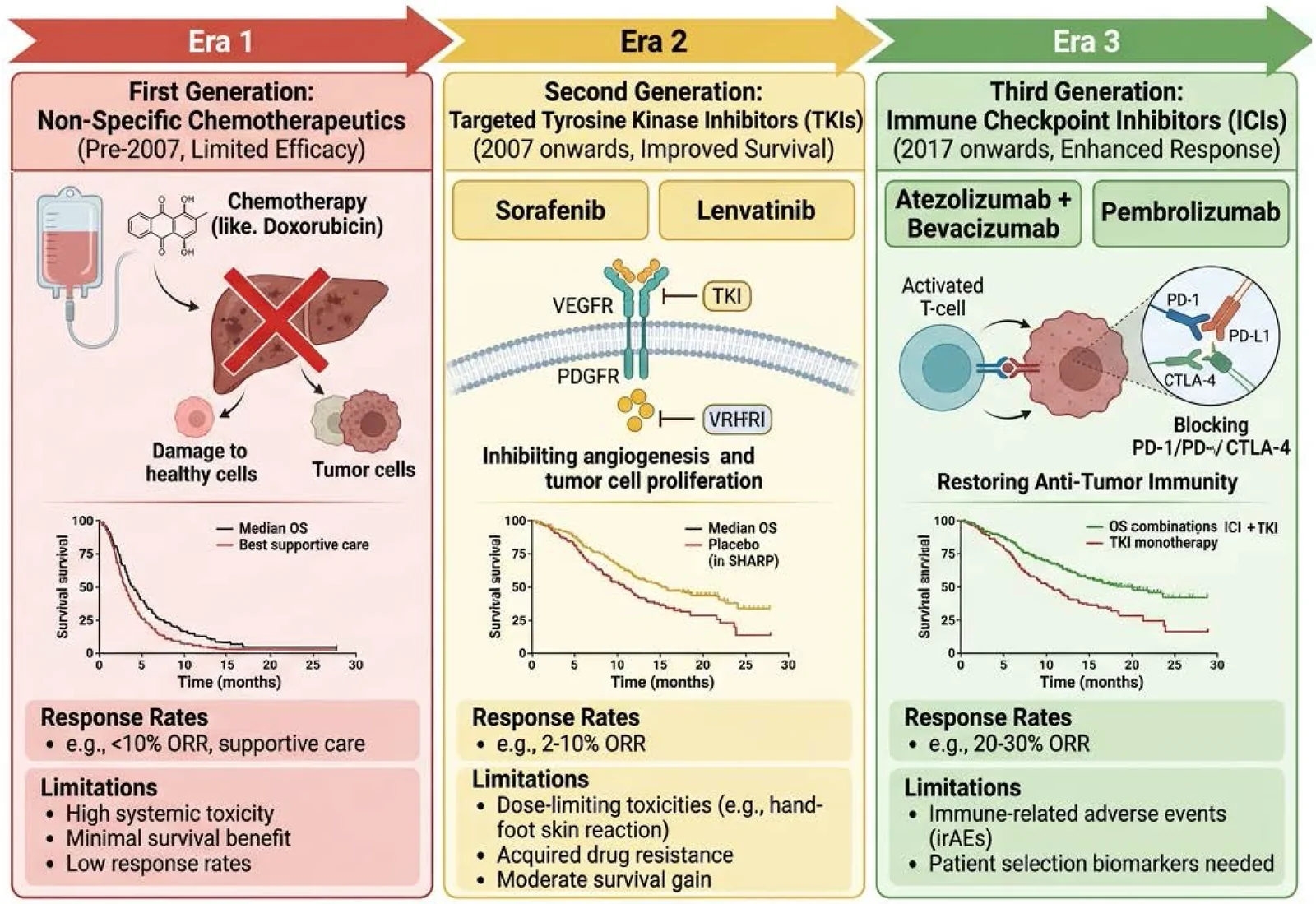
** Three Generations of Pharmacological Intervention in HCC.** This summary highlights the shift from non-specific chemotherapy to precision medicine and immunotherapy. It details the primary molecular mechanisms, representative clinical drugs, and the associated clinical outcomes for each era. While survival rates have significantly improved in the third generation (ORR 20-30%), Ongoing challenges include drug resistance in Era 2 and immune-related adverse events (irAEs) in Era 3.

**Figure 4:**
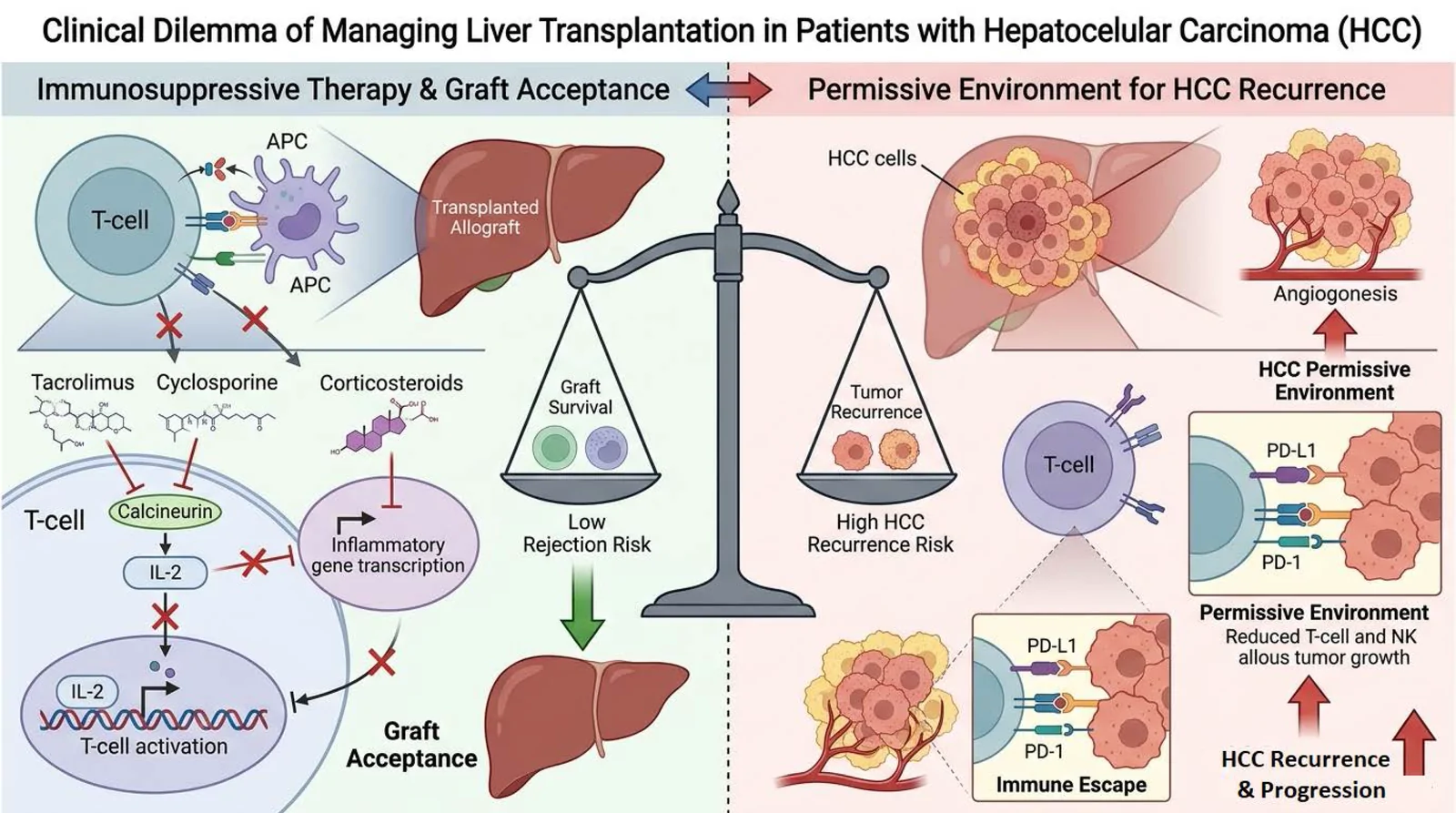
** The Interplay Between Graft Acceptance and HCC Recurrence Risk.** This figure highlights the dual-edged nature of post-transplant immunotherapy. While immunosuppression is mandatory to achieve graft acceptance and low rejection risk (left), it simultaneously impairs the body's anti-tumor surveillance. This creates an environment where residual HCC cells can undergo immune escape via PD-1 signaling and promote neoangiogenesis, significantly increasing the risk of tumor recurrence and progression (right).

The evolution of HCC treatments spans from chemotherapy only, to the era of molecular targeted drugs (sorafenib), and finally to modern combination immunotherapies (e.g., atezolizumab-bevacizumab) which are now standard first-line treatments, often combined with locoregional techniques (Figure 3). Liver transplantation (LT) is preferred treatment for patients with early-stage HCC who meet the Milan criteria or those successfully downstaged 17. LT removes the entire cirrhotic liver, theoretically eliminating the field of cancerization and the source of new tumors. However, the long-term success of LT is threatened by two major, interconnected challenges: post-transplant HCC recurrence and complications arising from immunosuppression (Figure 4) 63. The 5-year survival rates after LT for HCC range from 60-70%, but recurrence rates of 8-20% remain significant 18, 19.

To prevent allograft rejection, LT recipients require lifelong immunosuppression. The standard maintenance regimen typically involves calcineurin inhibitor (CNI) like tacrolimus or cyclosporine, antiproliferative agent like mycophenolate mofetil, and corticosteroids. While effective at preventing rejection, these drugs have profound implications for cancer control. Calcineurin inhibitors are associated with significant side effects including nephrotoxicity, neurotoxicity, diabetes, and hypertension 64. Crucially, CNIs promote carcinogenesis by increasing TGF-β production and potentially enhancing tumor cell invasiveness. Moreover, global immunosuppression compromises immune system's ability to surveil and eliminate micrometastases 65.

mTOR inhibitors such as sirolimus and everolimus block mTOR pathway and, unlike CNIs, possess anti-proliferative and anti-angiogenic properties. They have been associated with lower incidence of *de novo* malignancies and potentially reduced HCC recurrence 66. However, they can cause dyslipidemia, impaired wound healing, and mouth ulcers, and are not always suitable as primary monotherapy 67. Fundamental conflict is that effective graft preservation requires dampening immune system, while effective cancer control requires robust immune response to recognize and eliminate tumor cells 68.

### Immunotherapy Conundrum

The advent of ICIs like nivolumab (anti-PD-1) and atezolizumab (anti-PD-L1) has revolutionized advanced HCC treatment. By blocking brakes on T-cells, these drugs unleash potent anti-tumor immune response. Recent clinical trials have demonstrated significant survival benefits. The CheckMate 9DW trial showed that nivolumab plus ipilimumab significantly improved overall survival versus lenvatinib or sorafenib (median OS: 23.7 vs 20.6 months; HR 0.79), positioning this combination as an emerging first-line option, especially for patients who cannot receive bevacizumab 69.

However, use of ICIs in transplant context is fraught with danger. ICIs remove peripheral tolerance mechanisms preventing T-cells from attacking the graft. Multiple retrospective studies have shown that use of pre-transplant ICIs leads to acute allograft rejection rates as high as 25-40%, resulting in graft loss and patient mortality 70, 71. This risk is highest when interval between ICI administration and transplantation is short and immune-related adverse events occur, which indicate immune overactivation 70. The devastating consequence of graft rejection following ICI therapy limits the utility of these powerful agents in the transplant population 72.

For patients beyond Milan criteria, downstaging therapies are used to shrink tumors to qualify for transplant. While ICIs are effective downstaging agents, their use creates primed immune system that may attack new liver immediately after transplant. Current consensus suggests washout period, but this risks tumor progression. The IMbrave050 trial, which evaluated adjuvant atezolizumab plus bevacizumab versus active surveillance, initially showed improved recurrence-free survival, but updated analysis with longer follow-up no longer demonstrates sustained benefit, highlighting the complexity of adjuvant immunotherapy in HCC 73.

A critical clinical dilemma arises from these mandatory washout periods, which typically range from 4 to 12 weeks depending on the agent and institutional protocol. During this treatment-free window, patients face significant risk of unchecked tumor progression, potentially rendering them ineligible for transplantation. mTOR inhibitors offer a compelling alternative to traditional immunosuppressants in LT recipients, demonstrating non-inferiority in preventing graft loss while providing a safer profile than CNIs. Their primary advantage is the amelioration of CNI-induced renal impairment, alongside reduced rates of HCC recurrence and *de novo* malignancies. Their utility is frequently limited by dyslipidemia, impaired wound healing, mouth ulcers, and incomplete anti-tumor efficacy as monotherapy 74. Moreover, mTORC1 inhibition results in compensatory activation of PI3K-AKT, MAPK, and RAS signaling, a phenomenon that may paradoxically enhance tumor proliferation and tumor resistance 75. In addition, mTORC1 blockade predominantly inhibits cell growth without directly triggering apoptosis 76. These existing limitations underscore the urgent need for novel agents that can bridge this therapeutic gap, providing anti-tumor activity without increasing rejection risk.

### Need for Dual-Function Drug

The ideal pharmacological agent for HCC patients undergoing or awaiting liver transplantation would possess multiple critical properties: potent anti-tumor activity to effectively kill HCC cells or induce dormancy; immunomodulation without global suppression to prevent rejection without crippling anti-tumor immunity; low nephrotoxicity to spare kidneys; metabolic safety without exacerbating diabetes or dyslipidemia; and mechanistic synergy targeting core pathogenic drivers. Current drugs fail to meet all these criteria. CNIs are nephrotoxic and pro-oncogenic. mTOR inhibitors have better anti-cancer profiles but distinct side effects. ICIs are too risky for rejection. This gap in therapeutic arsenal highlights urgent need for novel agents with dual-function capability combining antitumor efficacy with favourable immunomodulatory profiles. Such an agent would represent paradigm shift in the management of HCC in the transplant setting 6, 77.

In addition to novel multimodal agents like CIGB-552, other strategies such as dual-targeted proteolysis-targeting chimeras (PROTACs) designed to simultaneously degrade previously “undruggable” targets are under investigation. Chen *et al*. 78 developed C116, a first-in-class MLKL degrader that effectively induces parthanatos, a form of programmed cell death, in HCC cells and demonstrated strong antitumor activity in orthotopic mouse models, positioning it as a promising therapeutic candidate for investigating MLKL's non-necroptotic functions. Concurrently, Wan *et al*. 79 reported RD12 as the first-in-class RNF4 PROTAC degrader, which selectively degrades RNF4 via the ubiquitin-proteasome system, induces DNA damage and apoptosis, and exhibits significant antitumor efficacy with no observable toxicity in HCC xenograft models. In the context of senescence and MASH driven HCC, Yang *et al*. 80 demonstrated that a BCL-xL/BCL-2 PROTAC effectively cleared senescent liver cells and reduces tumor burden in mice. Additionally, Zhang *et al*. 81 showed that targeting BET proteins with a PROTAC molecule elicits potent anticancer activity in HCC cells, further expanding the therapeutic landscape. Combination regimens pairing low-dose ICIs with mTOR inhibitors are also being explored to navigate this therapeutic gap 82. Each approach carries its own limitations: PROTACs face challenges in tissue-specific delivery and off-target effects, while ICI-containing combinations retain the inherent risk of triggering graft rejection.

## CIGB-552: Mechanism of Action and Therapeutic Rationale

### COMMD1 as the Central Target

CIGB-552 is a second-generation synthetic cell-penetrating peptide (CPP) developed by the Center for Genetic Engineering and Biotechnology (CIGB) in Cuba. It is derived from LALF_32-51_ region of Limulus anti-lipopolysaccharide factor, protein found in horseshoe crab lymph known for antimicrobial and anti-endotoxin properties 83. Through systematic alanine scanning and amino acid substitution, CIGB-552 was optimized for stability, cell penetration, and potent antitumor activity. The peptide effectively penetrates cells via both endocytic and transduction mechanisms 84-86.

**Figure 5:**
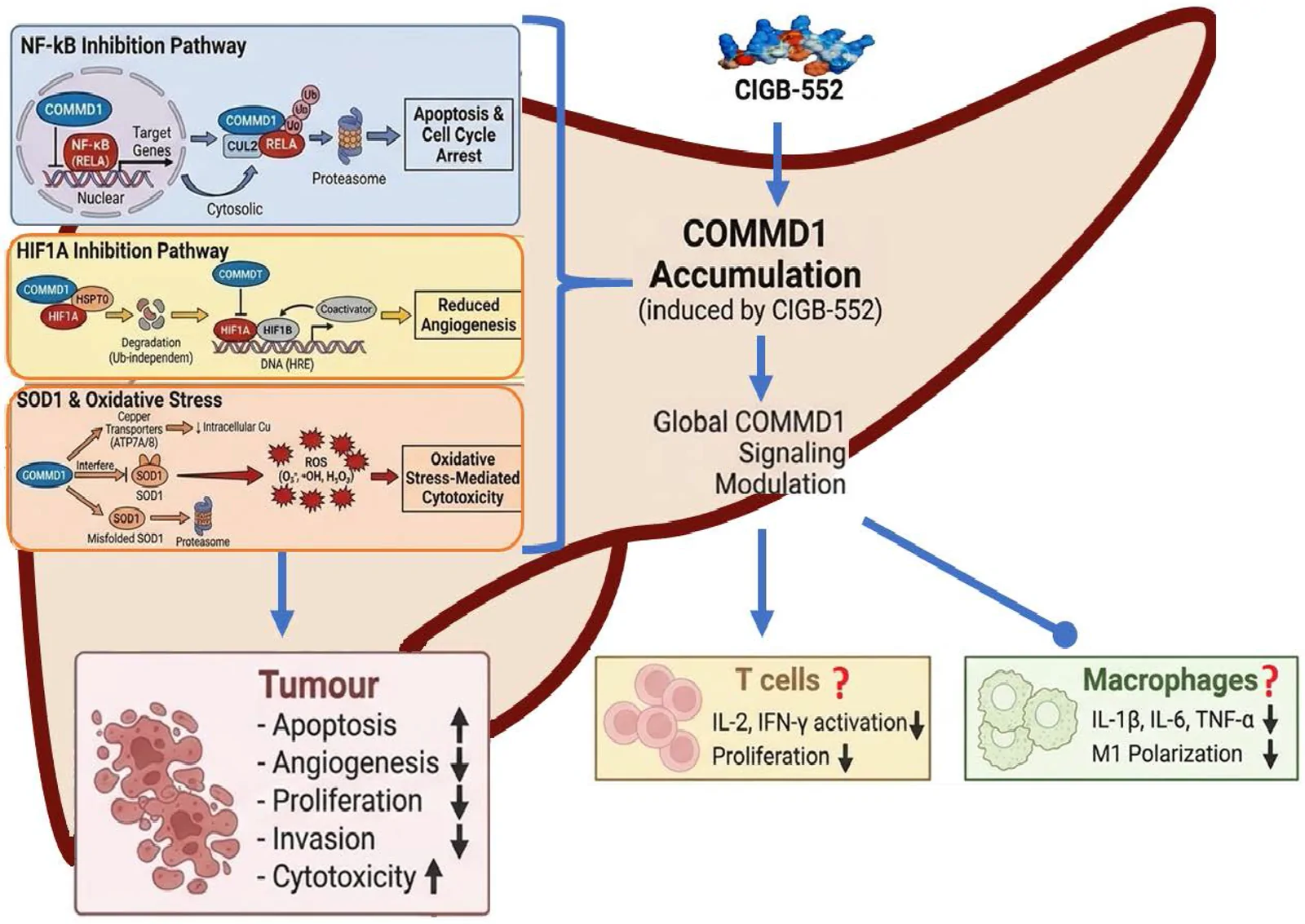
** Multimodal Anti-Tumor Activity of CIGB-552.** The modular illustration depicts the multi-target anti-tumor mechanisms of CIGB-552. Central to this pathway is the accumulation of COMMD1, which triggers three primary downstream cascades within tumor cells: (1) NF-κB inhibition pathway: COMMD1 regulates NF-κB signaling, leading to proteasome-mediated apoptosis and cell cycle arrest; (2) HIF1A inhibition pathway: Downregulation of HIF1A results in reduced angiogenesis; (3) SOD1 oxidative stress pathway: Inhibition of SOD1 induces ROS-mediated damage. Furthermore, the diagram illustrates the proposed modulation of the tumor immune microenvironment, highlighting the possible regulatory effects on T cells and macrophages, such as the decreased activation of IL-2 and IFN-γ.

The therapeutic potential of CIGB-552 lies in its multimodal mechanism of action, centred on the stabilization of the key regulatory protein COMMD1. COMMD1 is a 21 kDa multifunctional protein that serves as a critical node in various cellular processes, including copper homeostasis, endosomal sorting, and regulation of transcription factors 87, 88. Notably, COMMD1 is a negative regulator of NF-κB, HIF-1α signaling, and SOD1, positioning it as a unique target that can simultaneously impact the pathogenic pathways triad in HCC (Figure 5) 89-91.

In many cancers, including HCC, COMMD1 expression is downregulated, often correlating with poor prognosis 92. The protein XIAP (X-linked inhibitor of apoptosis protein) binds to and ubiquitinates COMMD1, targeting it for proteasomal degradation 93. Loss of COMMD1 removes critical brake on oncogenic signaling. CIGB-552 binds directly to COMMD1, probably preventing its interaction with XIAP and subsequent degradation. This leads to accumulation and stabilization of COMMD1 within cell, triggering cascade of antitumor effects that address multiple hallmarks of cancer simultaneously 94. The theoretical promise of CIGB-552 is supported by growing body of preclinical and clinical data. Studies in human lung (NCI-H460), colon (HT-29, HCT-116), and liver (HepG2, Huh7) cancer cell lines have demonstrated that CIGB-552 inhibits cell proliferation, induces G2/M cell cycle arrest, and triggers apoptosis 84, 85, 95. The peptide effectively penetrates cells via both endocytic and transduction mechanisms 86.

### Multimodal Mechanism: Disrupting Pathogenic Triad

The accumulation of COMMD1 induced by CIGB-552 results in multimodal attack on cancer cells, directly targeting NF-κB/HIF-1α/OS triad. First, regarding inhibition of NF-κB signaling by stabilizing COMMD1, CIGB-552 effectively shuts down NF-κB transcriptional activity. This downregulates expression of anti-apoptotic genes (Bcl-2, Bcl-xL) and pro-inflammatory cytokines (TNF-α, IL-6). The result is restoration of apoptotic sensitivity in cancer cells and reduction in pro-tumorigenic inflammatory microenvironment. CRISPR-Cas9 knockout of COMMD1 abolishes antitumor effects of CIGB-552, confirming COMMD1 as essential target for peptide's biological activity 96.

Second, concerning suppression of HIF-1α and anti-angiogenesis: COMMD1 is also a negative regulator of HIF-1α. It binds to HIF-1α and interferes with its ability to dimerize with HIF-1β, necessary step for DNA binding. COMMD1 recruits heat shock proteins (HSP70/HSP90) to HIF-1α, facilitating its proteasomal degradation in VHL-independent manner 91. CIGB-552-mediated COMMD1 stabilization leads to downregulation of HIF-1α target genes, including VEGF, PDGF, and GLUT1 96. Consequently, peptide exerts potent anti-angiogenic effect, starving tumor of blood supply, and disrupts metabolic adaptation that cancer cells rely on for survival in hypoxic niches 85, 96.

Third, regarding induction of oxidative stress via SOD1 inhibition, perhaps the most unique aspect of CIGB-552 is its ability to trigger lethal oxidative stress in cancer cells by indirect targeting SOD1. COMMD1 regulates copper export from the cell via ATP7A/B 97, and SOD1 is copper-dependent enzyme. By modulating copper availability, COMMD1 interferes with homodimerization of SOD1 subunits, final step in its maturation, and can target misfolded SOD1 for degradation 90. COMMD1 accumulation in CIGB-552 treatment leads to significant decrease in SOD1 activity, disabling cell's primary antioxidant defence mechanism 84. The result is a rapid accumulation of superoxide radicals and other ROS. This overwhelming oxidative stress causes lipid peroxidation, protein oxidation, and mitochondrial damage, triggering intrinsic apoptosis 84, 98. Importantly, cancer cells, which often operate under higher basal oxidative stress than normal cells, are selectively vulnerable to SOD1 inhibition, while normal cells equipped with robust compensatory antioxidant systems are spared 32. These three mechanisms are intricately linked and act synergistically: NF-κB inhibition removes survival signals and sensitizes cells to death; HIF-1α suppression cuts off angiogenic and metabolic support; and oxidative stress delivers the final lethal insult orchestrating a comprehensive multimodal attack on tumor cell viability.

While the direct pharmacological and preclinical data for CIGB-552 have predominantly originated from the developing institution (CIGB) and its international collaborative network, the underlying mechanism of action is supported by robust, independently validated biology. Consequently, while independent replication of CIGB-552-specific pharmacological profiles, particularly in HCC models remains an area for future investigation, the molecular pathways through which it operates are firmly established in the broader scientific literature 88-93, 97, 99.

### Preclinical and Clinical Evidence

A comprehensive summary of non-clinical and clinical evidence for CIGB-552 is presented in Table 1. In mouse xenograft models of human solid tumors, CIGB-552 administration significantly reduces tumor volume and increases survival 85, 100. Pharmacokinetic studies showed that the peptide was rapidly absorbed after subcutaneous injection, distributed widely, and cleared efficiently. Importantly, CIGB-552 did not cause cytotoxic effects in non-tumor cells, demonstrating selectivity for cancer cells. Toxicity studies in mice and dogs have shown excellent safety profile, with no signs of haematological, hepatic, renal, or cardiac toxicity at therapeutic doses 100, 101.

A first-in-human, open-label, 3+3 dose-escalation Phase I trial (ARGOS) evaluated CIGB-552 in 24 patients with refractory advanced solid tumors. CIGB-552 was administered subcutaneously as fixed total doses at 1.4, 2.8, 4.7, and 7.0 mg, given six times over two weeks. Overall, 298 adverse events were reported; toxicity was mild and manageable, with Grade 1-2 injection site pain (7.7%), asthenia (7.1%), and anorexia (6.0%) most frequent. Grade 3 adverse events comprised 19.5% (primarily at 7.0 mg), and 14.4% were serious (hospitalizations due to progression). The dose limiting toxicity was a Grade 3 pruritic rash in 3/7 patients at 7.0 mg, resolving within 30 minutes with antihistamines. No hematologic, hepatic, renal, or cardiac toxicity associated with the treatment occurred. The maximum tolerated dose was established at 4.7 mg three times weekly. Pharmacokinetic parameters correlated with preclinical data. Disease control rate at week 6 was 20%, with exploratory analysis suggesting enhanced overall survival in patients with pulmonary metastases 102.

Although this Phase I trial was completed years ago, subsequent clinical progress was hindered by the COVID-19 pandemic (2020-2022) and by the considerable financial and logistical barriers inherent to peptide drug development in developing nations. Efforts are now underway to initiate two Phase II studies.

## Rationale for CIGB-552 in Liver Transplantation

The unique pharmacological profile of CIGB-552 positions it as a theoretically promising candidate to address unmet needs in transplant oncology. By targeting NF-κB, HIF-1α, and SOD1, CIGB-552 attacks HCC through multiple independent pathways. This multimodal approach reduces likelihood of resistance development and is effective against heterogeneous tumor populations. Its ability to induce oxidative stress via SOD1 inhibition is particularly potent against cancer cells that have adapted to survive in high-ROS environments 84, 98. Administering CIGB-552 as adjuvant therapy post-transplant could eradicate micrometastatic disease that is the source of recurrence.

The anti-inflammatory properties of CIGB-552, mediated through NF-κB inhibition, are highly relevant to transplantation. Ischemia-reperfusion injury (IRI) immediately following reperfusion of the graft is a major source of early graft dysfunction and is driven by NF-κB-mediated inflammation 106-108. CIGB-552 may theoretically mitigate IRI. Furthermore, by modulating the immune response without causing global immunosuppression, CIGB-552 may prevent acute rejection while preserving anti-tumor immunity.

By inhibiting NF-κB, CIGB-552 may alleviate the sterile inflammation of IRI without inducing the global T-cell suppression characteristic of CNIs. Unlike tacrolimus and cyclosporine, which block calcineurin/NFAT signaling and broadly suppress T-cell activation, including both alloreactive and anti-tumor T-cell responses 109, 110, CIGB-552 selectively targets the NF-κB pathway. This selectivity means that while pro-inflammatory cytokine cascades are attenuated, antigen-specific T-cell recognition and cytotoxic function against residual tumor cells may remain largely intact. This potential dual-benefit mechanism, reducing IRI-driven inflammation while preserving immune surveillance, could represent one key advantage distinguishing CIGB-552 from traditional immunosuppressants that should be evaluated experimentally.

The absence of nephrotoxicity and neurotoxicity in preclinical and Phase I studies is a significant advantage over CNIs 100-102. Transplant patients often have compromised renal function due to cirrhosis and hepatorenal syndrome; adding non-nephrotoxic agent is crucial. The peptide's subcutaneous administration route is convenient for long-term management. For patients awaiting transplant, CIGB-552 could serve as bridge therapy. Unlike ICIs, which prime the immune system for rejection, CIGB-552 has an immunomodulatory rather than immunostimulatory profile.

Its use in the pre-transplant window could effectively reduce tumor burden without increasing the risk of post-transplant rejection. Preclinical studies have shown that CIGB-552 synergizes with chemotherapeutic agents like cisplatin, suggesting potential for combination regimens with TKIs or lower doses of mTOR inhibitors to enhance efficacy while minimizing toxicity 98. This synergy opens possibilities for multimodal treatment approaches that could further improve outcomes in HCC patients undergoing liver transplantation.

## Future Directions and Challenges

Currently, no direct preclinical or clinical evidence of CIGB-552 in HCC has been reported; its application in this context remains mechanistically rational but speculative, requiring empirical validation in transplantation models. The proposed immune tolerance mechanism is theoretical, and it remains uncertain whether COMMD1 stabilization might also impair necessary immune responses against the allograft. Preliminary experiments to support this hypothesis are in progress.

Key research priorities for translating CIGB-552 into the liver transplantation setting include: (1) Validation in orthotopic HCC liver transplant mouse models, which would provide the first direct evidence of CIGB-552's effects in the transplant setting and allow assessment of both anti-tumor efficacy and graft outcomes; (2) Assessment of pharmacological interactions with standard immunosuppressants, specifically calcineurin inhibitors (CNIs) such as tacrolimus and cyclosporine, as well as mTOR inhibitors such as sirolimus and everolimus, to determine whether CIGB-552 can be safely co-administered with existing immunosuppressive regimens; and (3) Evaluation of long-term safety and impact on graft survival, which is essential for any agent intended for use in transplant recipients requiring lifelong immunosuppression.

Given its ability to remodel the tumor microenvironment via NF-κB suppression and oxidative stress modulation, the sequential or combinational application of CIGB-552 with low-dose, low-potency immunotherapies prior to liver transplantation could be explored as a promising strategy to optimize the anti-tumor immune status without priming for severe alloreactive rejection. This approach would leverage CIGB-552's ability to create a more immunologically favorable milieu while minimizing the risk of overstimulating the immune system, though rigorous preclinical validation is required.

Establishing CIGB-552 as the standard of care in liver transplantation for HCC requires a phased approach. Phase II trials in HCC patients should stratify by etiology and molecular markers (COMMD1 expression, NF-κB activity) to identify responders and optimize patient selection. Initial studies could focus on the downstaging window, comparing CIGB-552 to transarterial chemoembolization or TKIs; later phases could evaluate it as a CNI-sparing maintenance regimen to preserve renal function and reduce recurrence risk. Pharmacodynamic biomarkers, such as serum cytokines (IL-6, TNF-α), oxidative stress markers (MDA, 8-OHdG), or circulating tumor DNA, could be employed to guide dosing and monitor drug activity. Combination strategies warrant investigation: CIGB-552 plus mTOR inhibitors may enhance anti-angiogenic effects, while pairing with belatacept or low-dose steroids could yield rejection-free, anti-cancer regimens.

Furthermore, pharmacoeconomic considerations merit attention. As CIGB-552 is developed by Cuba's Center for Genetic Engineering and Biotechnology, a region with a historical track record of producing cost-effective biologics, as exemplified by the world's first meningitis B vaccine, Heberprot-P for diabetic foot ulcers, and nimotuzumab for cancer treatment. This peptide may offer significant accessibility advantages in resource-limited regions where liver cancer is highly prevalent, such as sub-Saharan Africa, East and Southeast Asia, and parts of Latin America. While formal pharmacoeconomic analyses have not yet been conducted, the historical precedent of Cuba's biotechnology sector in producing affordable biologics provides a reasonable basis for anticipating accessibility advantages.

## Figures and Tables

**Table 1:** Summary of Preclinical and Clinical Evidence for CIGB-552

PHASE / RESEARCH FOCUS	EXPERIMENTAL MODELS	MAJOR FINDINGS AND PROPOSED MECHANISMS	LIMITATIONS
1. *In Vitro* Mechanistic & Cytotoxicity Studies 84, 86, 96, 103-105	Human lung (H460) and colon (HT-29, HCT-116, etc.) cancer cell lines; cancer cell line panel (various origens, including HCC); CRISPR-COMMD1 KO cells; 2D and 3D spheroid cultures; Yeast Two-Hybrid.	CIGB-552 selectively kills tumor cells regardless of molecular origen. COMMD1 was identified as the specific intracellular target. COMMD1 KO cells resist CIGB-552-induced death. COMMD1 stabilization → 1. Inhibition of NF-κB (increased RelA ubiquitination). 2. Inhibition of HIF-1α (anti-angiogenic). 3. Decreased SOD1 activity → increased ROS → mitochondrial apoptosis (Bax/Bcl-2 shift, caspase-3 activation).	Micromolar concentrations are required for cytotoxicity; *in vitro* models lack immune system participation; mechanisms validation rely heavily on KO models.
2. *In Vivo* Efficacy & Biodistribution (Preclinical) 85, 98, 100, 101	Syngeneic mouse models (TC-1 lung cancer in C57BL/6; CT-26 colon in BALB/c); Xenograft model (HT-29 in nude mice); Spontaneous tumors in pet dogs.	Significant tumor growth inhibition and increased survival. Anti-angiogenic effect (reduced microvessels). Synergy with Cisplatin (98% growth inhibition) and 5-FU. Rapid tumor uptake (20 min) and retention (24h). Safe in dogs.	Moderate efficacy at lowest doses in xenografts suggests dose intensification is needed; mouse models lack human tumor heterogeneity. No relevant models of HCC studied
3. Pharmacokinetics (PK) & Toxicology (Preclinical) 85, 100, 101	BALB/c mice, Sprague Dawley & Wistar rats, healthy and tumor-bearing.	Subcutaneous (s.c.) route is optimal for antitumoral activity. Rapid absorption (Tmax ~15-20 min). Half-life ~1.6h (rats). Repeated dose administration: MTD in mice: 30 mg/kg. Single dose administration in rats: 20 mg/kg causes unwanted side/toxic effects. Repeated dose administration in rats for 28 days: CIGB-552 has no detectable systemic toxicity in rats of both sexes up to the maximum dose of 3.2 mg/kg	Rapid systemic clearance requires frequent dosing. Renal accumulation at high doses indicates potential nephrotoxicity risk; local injection site reactions.
4. Clinical Translation (Phase I Human Trial) 102	Human patients (n=24 evaluable) with advanced, progressive solid tumors (breast, uterine, sarcoma, etc.) refractory to standard therapy.	MTD established at 4.7 mg. DLT was pruritic maculopapular rash (at 7 mg). PK showed rapid clearance (t1/2 ~0.9-3.3h). Primary efficacy signal was disease stabilization, notably improved survival in patients with lung metastases. No hematologic/ hepatic/ renal toxicity. The safety profile support further clinical development	Rapid systemic clearance requires frequent dosing. No HCC patients in the trial.

## Abbreviations

HCC: Hepatocellular carcinoma
NF-κB: nuclear factor-kappa B
HIF-1α: hypoxia-inducible factor-1α
NAFLD: non-alcoholic fatty liver disease
NASH: non-alcoholic steatohepatitis
MASLD: metabolic dysfunction-associated steatosis liver disease
TKIs: tyrosine kinase inhibitors
HBx: Hepatitis B virus X protein
ICIs: immune checkpoint inhibitors
COMMD1: Copper Metabolism MURR1 Domain-containing protein 1
ROS: Reactive oxygen species
LT: liver transplantation
HBV: hepatitis B virus
HCV: hepatitis C virus
CNI: calcineurin inhibitor
PROTACs: proteolysis-targeting chimeras
